# Why Are Product Prices in Online Markets Not Converging?

**DOI:** 10.1371/journal.pone.0072211

**Published:** 2013-08-28

**Authors:** Takayuki Mizuno, Tsutomu Watanabe

**Affiliations:** 1 National Institute of Informatics, Tokyo, Japan; 2 Graduate School of Economics, University of Tokyo, Tokyo, Japan; 3 The Canon Institute for Global Studies, Tokyo, Japan; Cinvestav-Merida, Mexico

## Abstract

Why are product prices in online markets dispersed in spite of very small search costs? To address this question, we construct a unique dataset from a Japanese price comparison site, which records price quotes offered by e-retailers as well as customers’ clicks on products, which occur when they proceed to purchase the product. The novelty of our approach is that we seek to extract useful information on the source of price dispersion from the shape of price distributions rather than focusing merely on the standard deviation or the coefficient of variation of prices, as previous studies have done. We find that the distribution of prices retailers quote for a particular product at a particular point in time (divided by the lowest price) follows an exponential distribution, showing the presence of substantial price dispersion. For example, 20 percent of all retailers quote prices that are more than 50 percent higher than the lowest price. Next, comparing the probability that customers click on a retailer with a particular rank and the probability that retailers post prices at a particular rank, we show that both decline exponentially with price rank and that the exponents associated with the probabilities are quite close. This suggests that the reason why some retailers set prices at a level substantially higher than the lowest price is that they know that some customers will choose them even at that high price. Based on these findings, we hypothesize that price dispersion in online markets stems from heterogeneity in customers’ preferences over retailers; that is, customers choose a set of candidate retailers based on their preferences, which are heterogeneous across customers, and then pick a particular retailer among the candidates based on the price ranking.

## Introduction

The number of internet users worldwide is 2.4 billion, constituting about 35 percent of the global population. The number of users has more than doubled over the last five years and continues to increase [1]. In the early stages of the internet boom, observers predicted that the spread of the internet would lead the retail industry toward a state of perfect competition, or a Bertrand equilibrium [2]. For instance, *The Economist* stated in 1990 that “[t]he explosive growth of the Internet promises a new age of perfectly competitive markets. With perfect information about prices and products at their fingertips, consumers can quickly and easily find the best deals. In this brave new world, retailers’ profit margins will be competed away, as they are all forced to price at cost” [3]. Even academic researchers argued that online markets will soon be close to perfectly competitive markets [4]–[6].

Has this prediction come true? Unfortunately not. Even now, e-retailers quote different prices for a particular product, and those who quote prices above the lowest price still survive in the market. This is reflected in empirical studies on a variety of products showing that a wide dispersion in the prices quoted by e-retailers can be observed [7]–[13]. An important implication of the existence of such a wide price dispersion is that customers do not make their purchase decisions on the basis of product prices alone [14]–[18]. If this is the case, the question arises: How do customers decide from which e-retailer to purchase a product? This is the main question we address in this paper. Specifically, we seek to answer this question by applying statistical methods to a unique dataset on online prices and transactions collected from a Japanese price comparison site.

The novelty of our approach is that we seek to extract useful information on the source of price dispersion from the shape of price distributions rather than focusing merely on the standard deviation or the coefficient of variation of prices, as previous studies have done. Another way in which our approach differs from those adopted in previous studies is that we pay attention both to sales prices (i.e., price quotes set by retailers) and to purchase prices (i.e., prices at which customers clicks occur), while previous studies have focused only on sales prices. This makes it possible for us to examine how retailers’ price setting behavior and customers’ purchasing behavior are interconnected with each other and lead to the emergence of price dispersion in online markets.

The rest of the paper is organized as follows. We first provide a description of the dataset employed in this paper. Next, we confirm the existence of substantial price dispersion on the price comparison website and then show that customers choose the retailer from which they purchase a product based on the price rank rather than the price difference across retailers. We present statistical regularities regarding the price rank at which customers purchase a product and the price rank at which retailers post their prices when they enter the market. It is showed that both the probability of purchase by customers and the probability of price posting by retailers declines exponentially with price rank, and that the exponents associated with them are almost identical. This suggests that the reason why some shops set prices at a level substantially higher than the lowest price is that they know that some customers will choose them even at that high price. We then calculate the conditional probability that a retailer with a particular attribute (e.g., accepting credit card payment) is clicked on and compare this with the unconditional probability that a retailer is chosen in order to estimate the contribution of that attribute. Applying this idea, we estimate the brand value of shops.

## Analysis

### 1 Data

The data used in this paper are compiled from Kakaku.com, a major Japanese price comparison website [19], which lists product prices quoted by almost 2,000 consumer electronics retailers. (The number as of March 8, 2012, when we compiled our data, was 1,689.) Users of this website can find the prices quoted by retailers on the website as well as information on various retailer characteristics, such as whether they accept credit card payment, whether they also have physical retail premises, and the address of their distribution center. Consumers visiting the Kakaku.com website can use this information to choose a retailer from whom to purchase a product and can then click a button on the website that says “Go to retailer’s check-out page.” Our dataset consists of the records of all prices offered by each retailer (a total of around 802 million records) and the history of customer clicks on the “Go to retailer’s check-out page” button (around 210 million records) for all products offered from October 1, 2010 to January 31, 2012. In this paper, however, we focus only on the records for 6,385 major products that were sold for more than six months during this period and that received more than 1,000 clicks. The total number of clicks in connection with these products is about 110 million, constituting 50 percent of the total customer clicks during the observation period.

### 2 Price Dispersion on Kakaku.com

**Figure 1:**
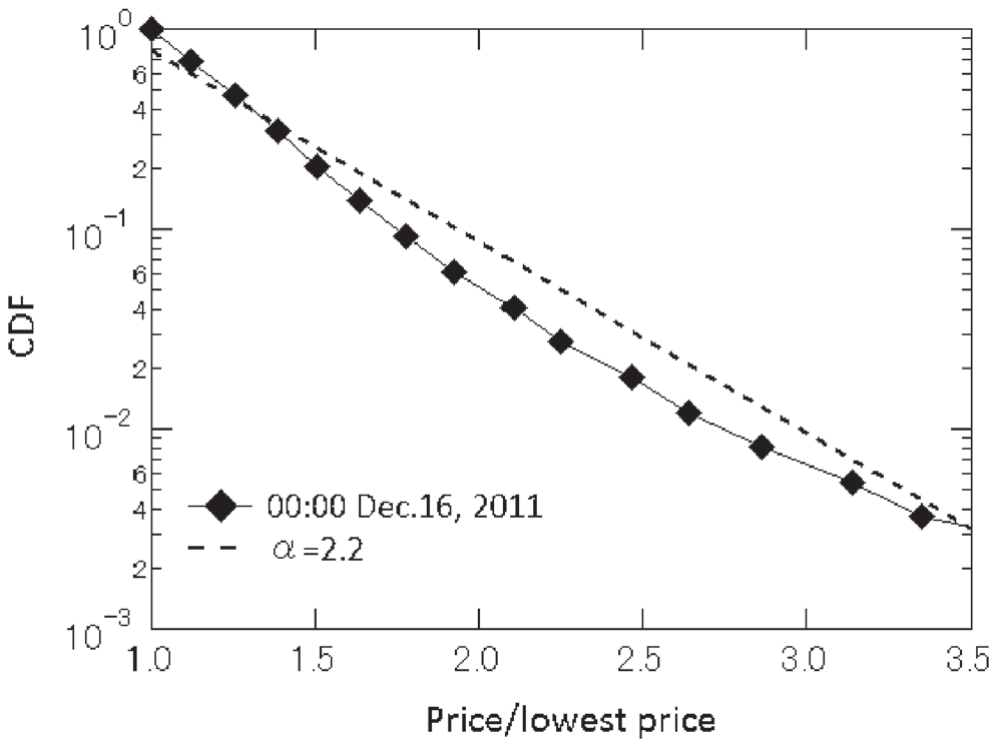
Cumulative distribution of prices divided by the lowest price. The series denoted by ♦ shows the distribution of price quotes available at 0∶00 on December 16, 2011, relative to the lowest price at that time. The dotted line is a reference line representing an exponential function with an exponent of 2.2.

Let us begin by examining price dispersion on Kakaku.com. The series denoted by ♦ in Figure 1 shows the cumulative distribution of price quotes 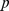 relative to the lowest price 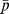 for each product available at 0∶00 on December 16, 2011. The tail of this distribution follows an exponential function of the form
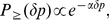
(1)where 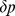 is defined as 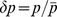 for each product and the estimate of the coefficient 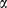 is 0.22. This figure shows that the fraction of retailers whose price quotes are more than 50 percent higher than the lowest price (i.e., 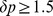) is about 20 percent, clearly indicating the presence of wide price dispersion. This result can be seen as further evidence against an important law in economics, the law of one price (LOP), which, as discussed extensively by [20], is also violated in a range of other markets.

**Figure 2:**
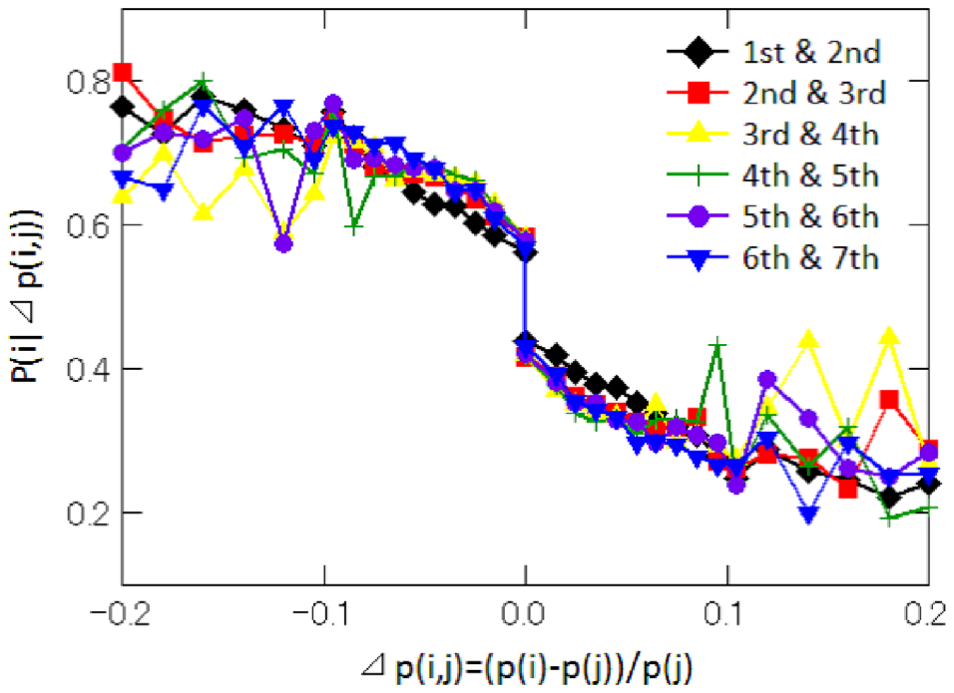
Relationship between the price gap and the probability that a retailer is clicked. The horizontal axis shows the price gap defined by 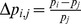, where 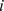 and 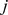 are two adjacent numbers. The vertical axis shows the probability that retailer 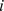 is clicked on, given that either 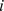 or 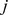 is clicked, i.e., 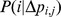. The series denoted by ♦(black), ▪(red), ▴(yellow), 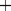
(green), •(purple), ▾(blue) represent the results for the combination of the first and second rank, the second and third rank, the third and the fourth rank, the fourth and the fifth rank, the fifth and the sixth rank, and the sixth and the seventh rank, respectively.

Next, we examine how customer clicks depend on the price gap between retailers. Specifically, we examine the relationship between the price gap between two retailers 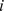 and 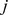 of successive ranks (e.g., the first and the second, the second and the third, etc.), which is denoted as 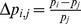, and the probability that retailer 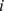 will be clicked on, given that either 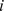 or 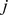 is clicked, 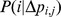. The result is shown in Figure 2 and, not surprisingly, indicates that the probability 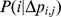 decreases the larger the price gap, 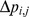, between two consecutively ranked retailers. However, it is worth noting that the relationship between 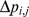 and the probability that a retailer is clicked is discontinuous at 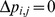. Specifically, when the price offered by retailer 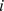 is only 1 yen lower than the price offered by retailer 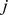, retailer 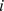 is able to obtain 60 percent of the total clicks. However, even if retailer 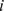 continues to reduce the price and quotes a price that is 10 percent lower than that of retailer 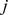, the fraction of clicks retailer 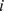 attracts increases only to about 70 percent. These results imply that customers choose a shop from which they purchase by focusing on the price rank gap between shops rather than on the simple price gap.

### 3 Customers’ Decision on where to Purchase

**Figure 3:**
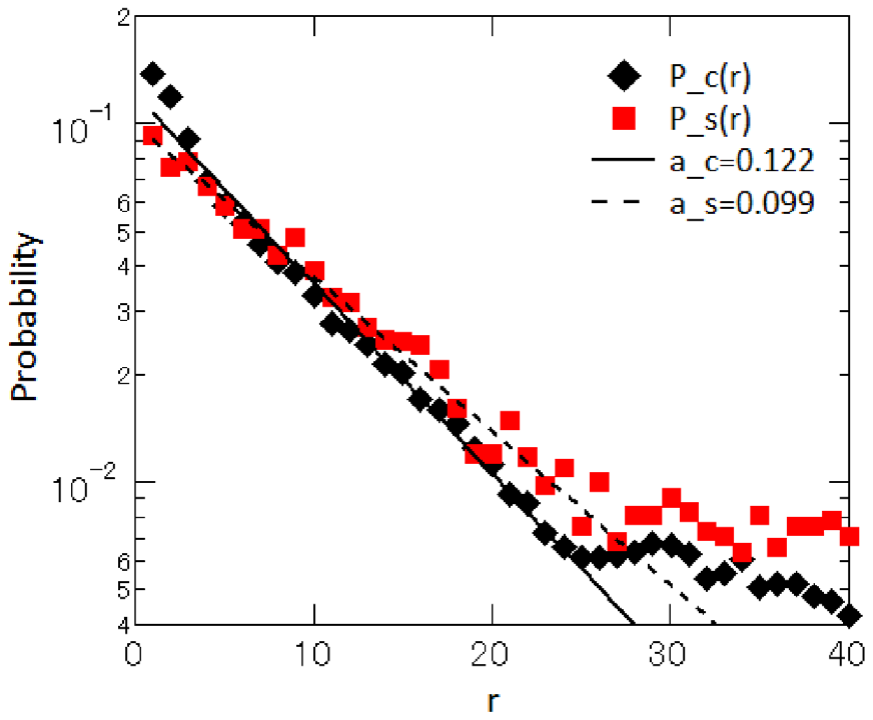
The probability that retailers post prices at rank 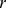 upon entry or re-entry to the market, 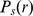, and the probability that customers click a retailer offering a price at rank 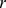, 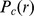. This figure is for the Sony Blu-ray disc recorder with the model number “BDZ-AT700.” The solid and dotted lines represent exponential functions with exponents of 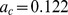 and 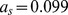, respectively.

In this section, we look at statistical regularities regarding the price rank at which customers click on the “Go to retailer’s check-out page,” as well as the price rank at which retailers post their prices when they enter the market. Figure 3 shows the relationship between the price rank of a retailer and the probability that customers click on that retailer for a specific product, namely the Sony Blu-ray disc recorder with the model number “BDZ-AT700.” The figure indicates that although the retailer offering the lowest price attracts the largest number of clicks, this falls far short of an overwhelming majority, and that the click probability of the retailer offering the tenth lowest price is not zero. The click probability for the first-ranked retailer (offering the lowest price) is about 14 percent, that for the second-ranked retailer (offering the second lowest price) is about 11 percent, and that for the tenth-ranked retailer is about 3.3 percent. This probability distribution is well approximated by the exponential function
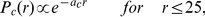
(2)where 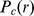 is the probability of being clicked at rank 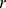, and 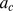 is a coefficient, which is estimated to be 0.122.

We show that the relationship between price rank 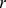 and click probability 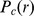 of the large majority of products that are sold by more than 20 retailers follows an exponential function. To do so, we approximate for each product the click probability 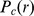 by a constant, a linear function, an exponential function, and a power law function, as follows:
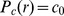


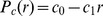


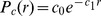


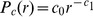
(3)where the coefficients 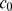 and 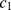 are estimated using the maximum likelihood method. We compare these four specifications using the Akaike Information Criterion (AIC) and the Bayesian Information Criterion (BIC) for each product and find that the exponential specification is chosen for 80.8 percent of all products, while the linear specification is chosen for 10.6 percent, and the power specification for 8.5 percent. To check the robustness of this result, we repeat this exercise using a dataset covering a different period, namely November 1, 2006 to September 30, 2007. We focus on 2,239 products that are sold by more than 20 retailers and obtain more than 1,000 clicks. We find that the exponential specification is chosen for 86.5 percent of the products, while the linear specification is chosen for 4.4 percent, and the power specification for 9.1 percent, indicating that the result does not depend on the sample period examined.

**Figure 4:**
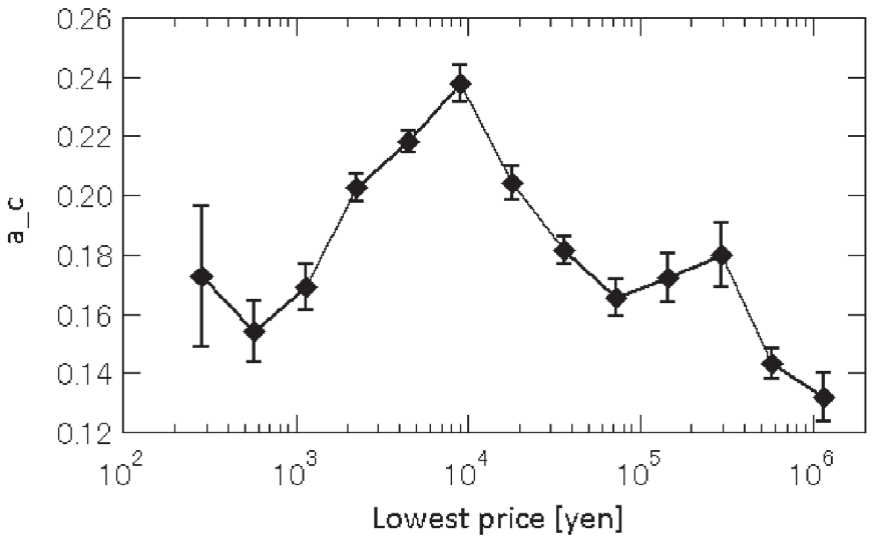
The estimated exponents 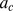 for different products. We split the entire sample of observed purchase prices into groups with different lowest prices at the time when clicks occurred, and then estimate 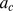 for each group.

Next, we propose a hypothesis to explain the observed relationship between price rank and click probability. We focus on the difference in customer preferences regarding various retailer attributes. For instance, a customer who wants to pay by credit card will choose a shop that accepts credit card payment. We assume that a customer first chooses a set of retailers which satisfy a certain set of criteria determined by the customer, and then purchases the product from the retailer offering the lowest price among them. Importantly, customers are assumed to be heterogeneous in terms of their preferences over shop attributes. That is, some customers may prefer shops that accept credit cards, while others may not prefer such shops. Given these assumptions, the probability that a retailer with rank 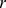 in terms of price is clicked is given by
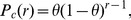
(4)where 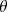 represents the probability that a particular retailer belongs to the set of favorite retailers for a customer. Equation 4 simply states that a retailer with rank 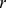 will be clicked only when none of the retailers offering a lower price are included in the set of favorite retailers. Comparing equations 2 and 4, we obtain 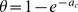. From this, we estimate that coefficient 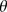 is 0.115. That is, when 100 retailers sell this product, the number of favorite retailers is only 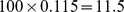. In other words, customers on average ignore 88.5 percent of retailers, including some or many that offer a lower price on the product the customer is interested in. Note that the coefficient 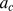 may differ across products. Figure 4 shows how the coefficient 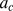 for each product depends on the lowest price quoted for that product. The figure indicates that there exists a convex relationship, with coefficient 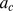 highest for prices in the range of 10,000 yen (or about 100 US dollars), implying that customers do not pay much attention to shop attributes when they purchase products in this price range and price competition therefore is fiercer for such products.

**Figure 5:**
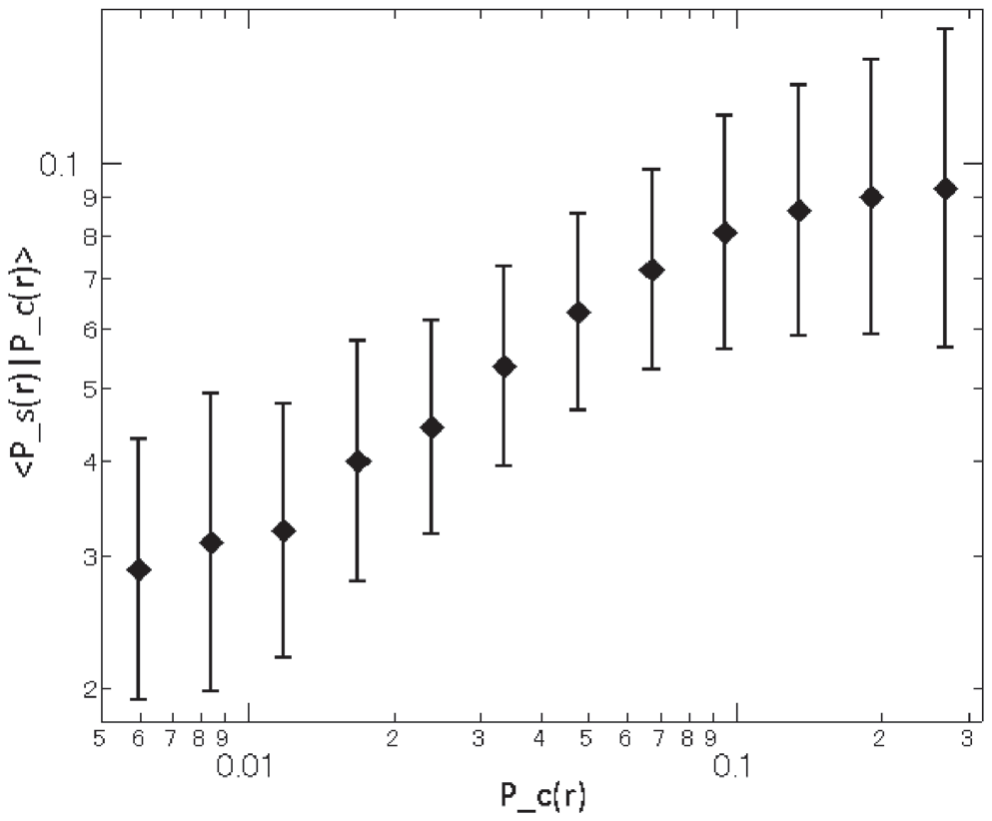
Relationship between the probability that customers click a retailer at rank 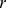, 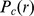, and the probability that retailers post prices at rank 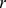, 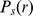.

Finally, we compare purchase prices (i.e., the price at which a customer clicked on the “Go to retailer’s check-out page” button) with sales prices (i.e., the price quoted by a retailer when it enters or re-enters the market) to make sure that price dispersion indeed stems from customers’ heterogeneous preferences over retailers. Note that, according to the rules set by Kakaku.com, retailers are not allowed to post prices when they have no inventory, so that retailers with no inventory must exit from the market until they have the item in stock again. The sales price refers to the price quoted by retailers either at the time of newly entering the market or at the time of re-entering the market. The series denoted by ▪(red) in Figure 3 shows how the probability that retailers post prices at rank 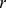 when they enter or re-enter the market, 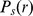, depends on price rank 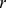. The relationship is an exponential function of the form
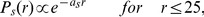
(5)which is similar to 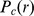 in equation 2. In fact, the exponents are 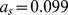 for sales prices and 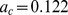 for purchase prices and thus are quite close to each other. To check whether this result holds for other products, we compare 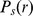 and 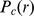 for all products sold by more than 20 retailers. The result is presented in Figure 5, which shows how the mean of 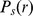 depends on the value of 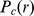. As shown in the figure, with a correlation coefficient of 0.65, these two probabilities are highly correlated, implying that retailers set a high price with a certain probability, because they recognize that customers click even at that high price with that probability.

### 4 Estimating Retailers’ Brand Value

**Figure 6:**
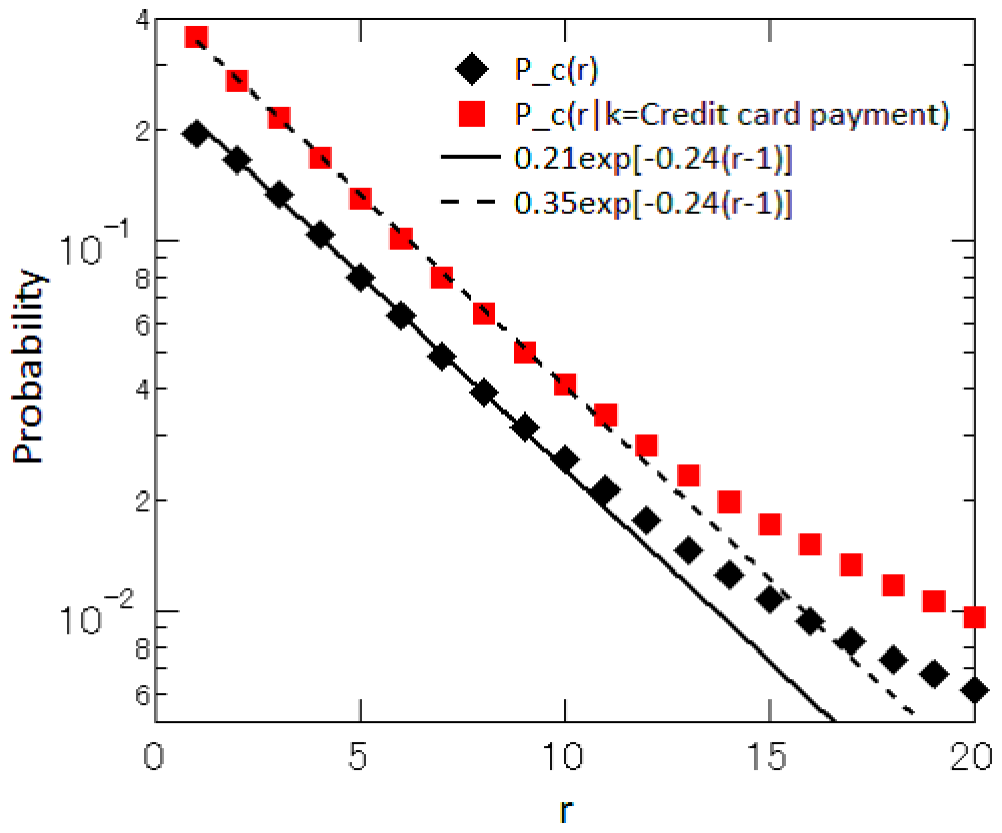
Probability of being clicked for shops accepting credit card payment. The probability of being clicked for shops accepting credit card payment, 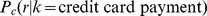, is denoted by ▪(red), while the unconditional probability, 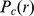, is denoted by ♦(black). The solid and dotted lines are reference lines with an exponent of 0.24.

In this section, we propose a method for estimating the brand value of a retailer by applying the line of reasoning regarding customers’ choice of retailer discussed in the previous section. Let 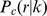 denote the probability that a retailer with a particular attribute 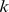 is clicked. We want to measure the value of this attribute. To do so, we employ the function 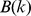, which is defined as follows:
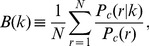
(6)where 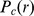 is the unconditional probability given in equation 2, and 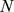 is the total number of retailers. Figure 6 presents the probability of being clicked for retailers that accept credit card payment, i.e., 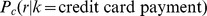, showing that the probability declines exponentially with 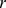, although the tail part deviates from a straight line. (We will come back to this issue later in this section.) Our estimate of 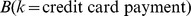 is 1.62, implying that the number of customers attracted by retailers accepting credit card payment is 1.62 times as large as the unconditional counterpart. We also find that 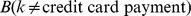 is 0.65, suggesting that retailers not accepting credit card payment attract 35 percent fewer customers than the average. We refer to 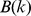 as the brand value of a particular attribute 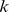.

We apply this method to various retailer attributes and the results are presented in Tables 1 and 2. Table 1 shows the results for the availability of various payment methods. For example, in the case of the option to send cash via registered mail, the difference between 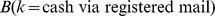 and 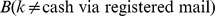 is very small, suggesting that it does not matter for customers whether a retailer offers to accept cash via registered mail. However, for other payment methods, such as collect on delivery, bank transfer, payment by credit card, payment at convenience stores, and financing, it matters considerably for customers whether such a payment method is available or not. It should be emphasized that the number of retailers accepting payment methods such as credit card payment, payment at convenience stores, or financing, is quite limited in this online market, as a result of which these retailers can attract more customers than other retailers.

**Table 1:** Estimates on brand value 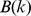.

Payment method *k*	Number of retailers	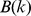 for retailers where	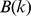 for retailers where
		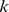 is available	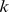 is not available
Collect on delivery	1,589	1.027	0.799
Bank transfer	1,262	1.070	0.783
Credit card	895	1.617	0.649
Payment at convenience stores	324	1.335	0.937
Financing	217	1.277	0.909
Cash via registered mail	80	0.954	1.002

Note: The total number of retailers is 1,662.

**Table 2:** Retailers’ location and estimated brand value.

Ranking interms of *B*(*k*)	Prefecture *k*	Number of shopsin prefecture *k*	Travel time to Tokyofrom prefecture *k*	Estimates on *B*(*k*)
1st	Gumma	21	1 h16 m	2.361
2nd	Kanagawa	96	0 h32 m	1.747
3rd	Ibaraki	11	1 h56 m	1.568
4th	Tokyo	556	0 h00 m	1.506
5th	Shizuoka	20	1 h26 m	1.302
29th	Ishikawa	10	3 h30 m	0.469
30th	Niigata	12	2 h39 m	0.409
31st	Nagasaki	5	4 h00 m	0.375
32nd	Yamaguchi	13	4 h09 m	0.313
33rd	Kagoshima	8	4 h00 m	0.288

Notes: 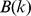 is calculated for prefectures that have more than five retailers, which is the case for 33 prefectures. The table shows the top five and the bottom five prefectures in terms of the estimates for 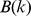. The total number of retailers is 1,662.

Table 2 shows the result for the geographical location of retailers. One might think that it does not matter for customers where retailers are located, because customers do not actually visit the retail premises and shipping is free. However, the results presented in Table 2 show that the value of 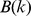 tends to be higher for retailers located in or near a major city like Tokyo, and lower for retailers located in prefectures far away from Tokyo. A possible reason is that customers may take into account the possibility that they have to visit the shop when serious problems arise.

Another factor that potentially affects the estimated brand value of retailers is the rating they receive from customers. Kakaku.com collects such ratings and displays a summary of those ratings as a percentage figure for each retailer. Specifically, the percentage figure, which is calculated every six months, shows the share of customers that responded that they would use the retailer again. Table 3 shows the estimated brand value for retailers with more than 80 percent positive ratings, those with 50 to 80 percent, and those with less than 50 percent. Note that in the table, we focus on about 700 retailers that have offered prices in the top twenty ranks. Of these, about 500 have been rated by customers, whether they are favorable or unfavorable, while the remaining 200 have not received any ratings. The table clearly shows that the estimated brand value tends to be high for retailers that have received ratings from customers than those that have not. The latter probably are retailers that fail to attract much attention from customers, and it is highly likely that they offer very low prices to survive in the market. Turning to retailers that have received customer ratings, the share of positive ratings appears to be positively correlated with the estimated brand value, although the correlation is very weak. Specifically, the brand value is 1.165 for retailers with more than 80 percent positive ratings, which is higher than the brand value for retailers with less than 50 percent positive ratings, but not very different from the brand value for retailers with 50 to 80 percent positive ratings. This weak correlation may be due to the limited number of customer ratings collected by Kakaku.com (even for retailers with at least one rating, the median number of ratings is only six).

**Table 3:** Customer ratings and estimated brand value.

Retailers with customer ratings		
	Number of retailers	Brand value
	492	1.142
Percentage of positive ratings		
Above 80 percent	374	1.165
Between 50 and 80 percent	60	1.169
Less than 50 percent	58	0.892
**Retailers with no customer ratings**		
	**Number of retailers**	**Brand value**
	204	0.669

Note: The brand value is estimated for 696 retailers that offer prices in the top 20 ranks.

**Figure 7:**
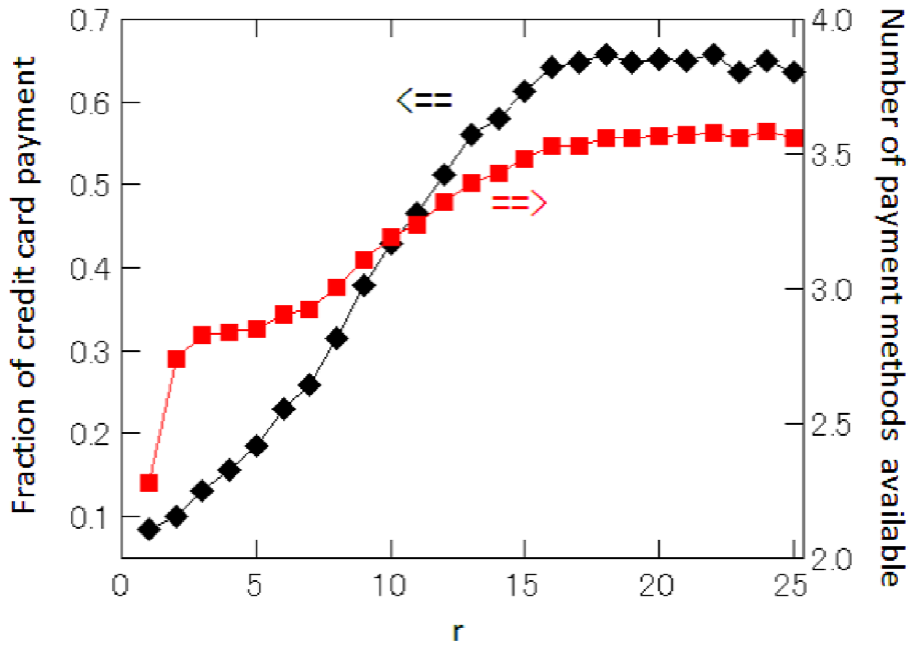
Fraction of retailers that accept credit card payment, and the average number of payment methods available at each retailer. The fraction of retailers that accept credit card payment is denoted by ♦(black), while the average number of payment methods available at each retailer is denoted by ▪(red).

As mentioned before, the tail parts of 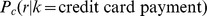 and 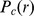 in Figure 6 deviate upward from a straight line, which suggests that retailers with a large 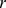 may possess a number of attributes that are attractive to customers. In order to see whether this is true or not, we look at how the fraction of retailers accepting credit card payment is related to the price rank, which is shown by the series denoted by 
(black) in Figure 7. The figure suggests that retailers offering lower prices are less likely to accept credit cards. It can also be seen that the probability that a retailer accepts credit cards monotonically increases until the 15th price rank. Next, we repeat the exercise, but now change the definition of 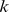 to include a variety of payment methods, i.e., 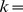 collect on delivery, bank transfer, payment by credit card, payment at convenience stores, and financing. The result is depicted by the series denoted by (red) in Figure 7, which indicates again that retailers offering the lowest prices tend to not accept a wide variety of payment methods.

Finally, we estimate the brand value of each retailer by calculating the conditional probability 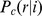, where 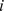 represents retailer 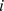. The highest brand value among all the retailers, 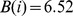, is recorded by a famous giant e-retailer known for offering a wide variety of products. We also find that some of the retailers with a high brand value are specialized in certain product categories such as wristwatches, air-conditioners, or in-car products. In contrast, shops with a small 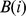 tend to be of small scale, and lack their own website and sell products only in online markets such as Yahoo, Amazon, and so on. In fact, the fraction of retailers without their own website is closely related with the value of 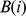; that is, the fraction of retailers without their own website is 29 percent for shops with 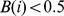, 7 percent for 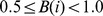, and 1 percent for 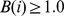.

## Results and Discussion

In this paper, we established three empirical facts. First, we showed that prices quoted by retailers on a price comparison website, where search costs are negligibly small, show considerable dispersion. We also showed that customers click on the link to a retailer’s website even if that retailer quotes a price that is substantially higher than the lowest price, although the probability that such a retailer’s link is clicked is smaller than that for the retailer offering the lowest price. Our second finding is that customers choose a retailer based on the price rank rather than the simple difference in quoted prices. For example, whether a retailer offers the first or the second lowest price matters, but the difference in yen between those two prices does not matter. Third, comparing the probability that customers click on a retailer with a particular rank and the probability that retailers post prices at a particular rank, we showed that both decline exponentially with price rank and that the exponents associated with the probabilities are quite close. This suggests that the reason why some retailers set prices at a level substantially higher than the lowest price is that they know that some customers will choose them even at that high price. Based on these findings, we proposed the hypothesis that price dispersion in online markets stems from heterogeneity in customers’ preferences over a variety of retailer attributes. Put differently, some attributes of retailers, such as the payment methods they accept, their warranty policy, and their reputation, enter customers’ utility function, which is heterogeneous across customers, so that the price is not the sole variable that determines consumption choices. In fact, we showed that retailers accepting a wide variety of payment methods, such as credit card payment and collect on delivery, tend to attract more customers than retailers accepting a limited number of payment methods, and tend to sell products at higher prices.
